# A comparison of the efficacy and safety of traditional Chinese medicine in preconditioning patients with diminished ovarian reserve that would undergo In Vitro fertilization: A network meta-analysis protocol

**DOI:** 10.1097/MD.0000000000024408

**Published:** 2021-01-29

**Authors:** Xiuyun Qin, Yongping Gong, Feng Yu, Jiangquan Song, Shuangqian Dong, Ruoqian Zhang, Jianwei Zhang

**Affiliations:** a The First Clinical, Shandong University of Traditional Chinese Medicine; b The Affiliated Hospital of Shandong University of Traditional Chinese Medicine; c Rizhao Maternal and Child Health Care Hospital, Rizhao, Shandong Province, China; d Rizhao Central Hospital.

**Keywords:** diminished ovarian reserve, IVF, meta-analysis, precondition, TCM

## Abstract

**Background:**

Diminished Ovarian Reserve (DOR) is a common disease in reproductive-age women in the diagnosis and treatment of infertility. The incidence of DOR increased quickly in recent years and had became one of the most important factors that made the quality of women life decline. Gynecology and reproductive medicine professors have made great efforts to explore good treatment methods all over the world. Traditional Chinese Medicine has made some achievement in treating DOR as a kind of complementary and alternative therapy In Vitro Fertilization (IVF) in recent years, it is indispensable to propose a network meta-analysis (NMA) protocol to discuss the efficacy and safety of TCM in IVF.

**Methods:**

A literature search will be conducted in 8 electronic databases.

**Results:**

The efficacy and safety of traditional Chinese medicine in preconditioning patients with diminished ovarian reserve that would undergo In Vitro Fertilization will be evaluated.

**Results:**

The systematic review will evaluate the efficacy and safety of TCM in IVF.

**Conclusion:**

The result of this study will provide reliable evidence of the use of TCM in IVF.

**INPLASY registration number:**

INPLASY2020110062.

**Ethics and dissemination:**

This review does not require ethical approval.

## 1 Introduction

Ovarian reserve plays a crucial role in reproductive-age women. It is used to predict prognosis as a function growing follicle number and the reproductive potential of harvested oocytes. Diminished ovarian reserve (DOR) affects approximately 10% of women seeking fertility treatment.^[1]^ DOR is characterized by poor fertility outcomes even when assisted reproductive techniques (ART) are used and represents a major challenge in reproductive medicine.^[2]^ The definition of DOR comes from European Society of Human Reproduction and Embryology (ESHRE) guideline in 2016 was:

1. age < 40, over 1 year normal menstrual cycle;
2. DOR symptoms;
3. abnormal ovarian reserve test.^[3]^

DOR is the early stage of premature ovarian failure (POF),^[4]^ they are associated to recurrent miscarriage, unexplained infertility, repeated planting failure in IVF/ICSI, preeclampsia and many other fertility problems.^[5,6]^

Kate Devine^[7]^ conducted a retrospective analysis of all autologous SART CORS cycles in 2004 and 2011 and found that DOR prevalence increased from 19% to 26% from 2004 to 2011. Compared with other infertility diagnoses, DOR patients may show poor responsiveness to gonadotropin stimulation, serious consequences beyond refractory infertility and has been shown to predict early menopause in several series,^[8–10]^ they also experienced decreased responses to ovulation induction, require higher doses of gonadotropin, higher IVF cycle cancellation rates and experience lower pregnancy rates.^[11,12]^

The function and regulation of DOR are still poorly understood now. As infertility populations in the world rapidly aging, the treatment of DOR has attracted more and more attention all over the world. The current treatment strategies for DOR is hormone replacement, HRT is the most commonly used therapy and Dehydroepiandrosterone (DHEA) has been reported to improve pregnancy chances with DOR,^[13,14]^ about one third of all IVF centers world-wide and many prospectively randomized trial are ongoing in many clinical practices.^[15]^ Treatment of infertility when the cause is limited to decreased ovarian reserve is empirical at present except for oocyte donation.^[16]^

In recent years, Traditional Chinese medicine (TCM)^[17–22]^ have made some progress in improving the outcome of IVF in those patients that were diagnosed with DOR. It is of some academic value to make a meta-analysis to make people more clear about the effectiveness and safety of traditional Chinese medicine in preconditioning patients with diminished ovarian reserve that would undergo In Vitro Fertilization.

## 2 Materials and methods

### 2.1 Study registration

We compliant preferred reporting items for systematic review and meta-analysis protocols guidelines to conduct this study.^[23]^ Our protocol of this NMA was registered on the INPLASY international platform of registered systematic review and meta-analysis protocols (ID = INPLASY2020110062). The DOI number is 10.37766/inplasy2020.11.0062, https://inplasy.com/inplasy-2020-11-0062/.

#### 2.1.1 Type of studies

Published randomized controlled trials (RCTs), Case-control studies and cohort studies evaluating the role of TCM (Not acupuncture) in treating women with DOR that would or had taken in IVF will be eligible for inclusion.

#### 2.1.2 Type of participants

Women of reproductive age with diminished ovarian reserve included, they also meets the diagnostic criteria of infertility of traditional Chinese medicine. All the participants were undergoing or due to accept IVF.

#### 2.1.3 Type of interventions

In this analysis, Chinese medicine decoction or Chinese patent medicine will be used as intervene method for assistant therapies in control group. Studies of acupuncture, massage and other external treatments will be excluded.

#### 2.1.4 Type of outcome measures

The primary outcomes will be changes in antral follicle count (AFC) and pregnant rate. The secondary outcomes will be improvement in basal serum FSH level (bFSH), basal serum E_2_(bE_2_), FSH/LH and AMH.

### 2.2 Exclusion criteria

We will exclude studies in which DOR was induced by unilateral or bilateral ovary removal surgery, chromosome abnormality, participants included which were diagnosed with premature ovarian failure (POF) or primary ovarian insufficiency (POI) and those studies without clear diagnostic criteria. IVF intervenes or full texts will also be considered ineligible for this analysis.

### 2.3 Database and search strategies

A comprehensive literature search will be conducted in 3 English and 5 Chinese electronic databases, including the Cochrane library, PubMed, EMBASE, WanFang Database, China National Knowledge Infrastructure, VIP Database, Chinese Biomedical Database and Chinese Clinical Trial Register to Search papers published in recent 5 years. We will apply a combination of Medical Subject Heading (MeSH) and free-text terms to implement search strategies. Table 1 shows details of the search strategy for PubMed.

**Table 1:** Detail of the search strategy for PubMed.

No.	Search item
#1	Medicine, Chinese Traditional [MeSH Terms]
#2	Medicine, Chinese Traditional [Title/Abstract] OR Traditional Chinese Medicine [Title/Abstract] OR Traditional Medicine, Chinese [Title/Abstract] OR Zhong Yi Xue [Title/Abstract] OR Chinese Traditional Medicine [Title/Abstract] OR Chinese Medicine, Traditional [Title/Abstract] OR Drugs, Chinese Herbal [Title/Abstract] OR Medicine, Tibetan Traditional
#3	#1 OR #2
#4	Ovarian Reserves [MeSH Terms]
#5	Ovarian Reserves [Title/Abstract] OR Reserve, Ovarian [Title/Abstract] OR Reserves, Ovarian [Title/Abstract] OR Reserve, Ovarian
#6	#4 OR #5
#7	In Vitro Fertilization [MeSH Terms]
#8	In Vitro Fertilization [Title/Abstract] OR Fertilization in Vitro [Title/Abstract] OR In Vitro Fertilizations [Title/Abstract] OR Test-Tube Fertilization [Title/Abstract] OR Fertilization, Test-Tube [Title/Abstract] OR Fertilizations, Test-Tube [Title/Abstract] OR Test Tube Fertilization [Title/Abstract] OR Test-Tube Fertilizations [Title/Abstract] OR Fertilizations in Vitro [Title/Abstract] OR Test-Tube Babies [Title/Abstract] OR Babies, Test-Tube [Title/Abstract] OR Baby, Test-Tube Test [Title/Abstract] OR Tube Babies [Title/Abstract] OR Test-Tube Baby [Title/Abstract] OR in-vitro activation
#9	#7 OR #8
#10	randomized controlled trial [Publication Type]
#11	controlled clinical trial [Publication Type]
#12	Randomized [Title/Abstract]
#13	Randomly [Title/Abstract]
#14	#10 OR #11 OR #12 OR #13
#15	#3 AND #6 AND #9 AND #14

#### 2.3.1 Study selections

Two researchers will independently undertake the search. All studies will be identified from electronic searches and imported into Endnote. Literature management software (X9) will first screen and duplicates will be eliminated. Evidently irrelevant studies will be excluded by reading the title and abstract of all articles, then the full texts of the remaining studies will be retrieved and assessed again. Advice from a third reviewer will be sought whenever disagreement arises between the 2 researchers.

#### 2.3.2 Data collection and management

Two of us will designe the data extraction sheet and abstract data independently from individual studies. Information concerning the participants, interventions, outcomes will be extracted from each study, they will appraise the quality of studies included, and disagreements will be resolved by discussion and consensus with professor Zhang.

### 2.4 Assessment of risk of bias in included studies

Two of us will assess the quality of included studies independently using the Cochrane risk of bias assessment tool. The results include the following domains: random sequence generation, allocation concealment, blinding of participants, blinding of outcome assessors, incomplete outcome data, selective reporting, and other sources of bias. An assessment of risk of bias will be made for the eligible studies based on the following 3 levels: “low risk of bias” “unclear risk of bias” “high risk of bias” by using information identified from the published articles and by contacting the authors when needed.

### 2.5 Statistical analysis

We will use Review Manager 5.3, provided by the Cochrane collaborations, for data analysis. We will assess heterogeneity across trials to decide whether it is meaningful to have the data pooled by using the *I*^2^ statistic. *I*^2^ ≦ 50% means moderate heterogeneity, it will be accepted; *I*^2^ > 50% means notable heterogeneity, original studies will be reviewed to check the raw data and the methods used were correct and identify some possible causes in that case, A random-effect model will be used if the variation could not be explained. If quantitative synthesis is not appropriate, we will use qualitative analysis.

### 2.6 Subgroup analysis

We will conduct subgroup analysis to explore the sources of heterogeneity if it is necessary.

## 3 Discussion

The quality of women's lives is of great significance to families and society. It is one of the hot spots in modern medical research to solve the fertility requirement of women with decreased ovarian reserve. We should summarize the existing research results timely and seek new directions. Treatments based on differentiation of syndromes are key to the theory of TCM. Differentiation of syndromes should be known by all accoucheurs, then more and more RCTs will appear and we may find more and more convenient, efficient, safe and economical ways to DOR.

## 4 Limitations

Articles published in languages other than English and Chinese will not be included in the study, but this will do not make a significant impact on this study.

## Author contributions

**Conceptualization:** Xiuyun Qin, Yongping Gong, Feng Yu.

**Data curation:** Xiuyun Qin, Yongping Gong, Feng Yu, Shuangqian Dong, Ruoqian Zhang.

**Formal analysis:** Shuangqian Dong.

**Funding acquisition:** Jianwei Zhang.

**Methodology:** Xiuyun Qin, Yongping Gong, Shuangqian Dong.

**Resources:** Jiangquan Song.

**Software:** Shuangqian Dong.

**Supervision:** Feng Yu, Jiangquan Song, Jianwei Zhang.

**Writing – original draft:** Xiuyun Qin, Yongping Gong.

**Writing – review & editing:** Xiuyun Qin, Ruoqian Zhang, Jianwei Zhang.

## Abbreviations

Abbreviations: AFC = antral follicle count, ART = assisted reproductive techniques, bE_2_ = basal serum E_2_, bFSH = basal serum FSH level, DOR = Diminished Ovarian Reserve, IVF = In Vitro Fertilization, MeSH = Medical Subject Heading, NMA = network meta-analysis, POF = premature ovarian failure, POI = primary ovarian insufficiency, RCTs = randomized controlled trials, TCM = Traditional Chinese Medicine.
